# PEGylated lipid nanocarrier for enhancing photodynamic therapy of skin carcinoma using curcumin: in-vitro/in-vivo studies and histopathological examination

**DOI:** 10.1038/s41598-020-67349-z

**Published:** 2020-06-26

**Authors:** Doaa A. Abdel Fadeel, Rabab Kamel, Maha Fadel

**Affiliations:** 1 Pharmaceutical Technology Unit, Department of Medical Applications of Laser, National Institute of Laser Enhanced Sciences, Cairo University, Giza, Egypt; 2 Pharmaceutical Technology Department, National Research Centre, Cairo, Egypt

**Keywords:** Drug discovery, Drug development, Cancer, Skin cancer

## Abstract

The use of (PEG)-grafted materials has a positive impact on drug delivery. In this study we designed PEGylated lipid nanocarriers (PLN) loaded with curcumin (Cur) to target skin cancer by photodynamic therapy. Cur is a polyphenolic compound having vast biological effects masked due to its low aqueous solubility. PLN were prepared using Tefose 1500 with different surfactants. PLN3, containing Tween 80, had the smallest particle size (167.60 ± 15.12 nm), Z = − 26.91 mV and, attained the highest drug release (Q24 = 75.02 ± 4.61% and Q48 = 98.25 ± 6.89%). TEM showed spherical, well-separated nanoparticles. The dark and photo-cytotoxicity study on a human skin cancer cell line (A431) revealed that, at all tested concentrations, the viability of cells treated with PLN3 was significantly lower than those treated by Cur suspension and, it decreased upon irradiation by blue light (410 nm). The amount of Cur extracted from the skin of mice treated by PLN3 was twice that of mice treated by aqueous drug suspension, this was confirmed by the increase in fluorescence intensity measured by confocal laser microscopy. Histopathological studies showed that PLN3 could extend Cur effect to deeper skin layers, especially after irradiation. This study highlights the possible efficacy of curcumin-loaded PEGylated lipidic nanoparticles to combat skin cancer by photodynamic therapy.

## Introduction

Photodynamic therapy (PDT) is a modality of cancer treatment that relies on a photochemical reaction between a compound called (photosensitizer) and a light source of a specific wavelength in the presence of oxygen. Photosensitizers are typically nontoxic, however, they are excited upon exposure to light of specific wavelength resulting in the production of either free radicals (type I reaction), or reactive oxygen species (ROS) (type II reaction) causing massive destruction of the tumor tissues^1^. The effect of the generated ROS is mainly confined to the tissues in the vicinity of the irradiation site. Consequently, if the tumor tissues are exclusively exposed to light, the distant healthy tissues can be kept unaffected^2^.

PDT has many advantages compared to chemotherapy and radiotherapy due to low invasiveness and systemic toxicity, decreased drug resistance, and high selectivity^3^. Consequently, PDT has been studied as a promising modality for the treatment of different types of skin cancers^4^. The success of PDT depends on the adequate choice of the photosensitizer and the corresponding light source. Light sources with short wavelengths, as blue light (400–450 nm), have short depth of penetration, so they can be used only for superficial skin lesions. Blue light has been used successfully in treating some types of skin cancers as basal cell carcinoma^5^ and melanoma^6^. Topical application of the photosensitizer on the desired site of action can avoid the severe cutaneous photosensitivity caused by their systemic use^7^.

The use of an effective and safe photosensitizer originating from a natural source, and the enhancement of its dermal delivery can contribute in the success of the photodynamic therapy.

Curcumin (Cur) is a polyphenolic compound derived from turmeric root with vast biological effects comprising photocytotoxicity upon excitation by blue light^8,9^. Unfortunately, these effects are limited by its low aqueous solubility and low stability. The selection of a suitable nanocarrier for Cur is essential to overcome these limitations.

Lipoid nanocarriers have attracted many researchers for enhancement of the dermal application of plant-derived drugs with low aqueous solubility^10–12^ because of the increase of drugs chemical stability, solubilization, topical film formation, increased skin hydration and occlusion, enhancement of skin penetration and nanoparticles physical stability. In addition, some recent studies have proved the capability of these nanocarriers to be deposited within the skin layers^10,11^, this can provide a beneficial advantage in the current study allowing for skin targeting and localized cytotoxic effect.

On another hand, poly(ethylene glycol) (PEG)-grafted biomaterials have great importance, this technology has a positive impact in drug delivery^13^. Based on this strategy, this study was oriented to the use of the PEGylated Tefose 1500 (mixed PEG-6 stearate and PEG-32 stearate) as the lipid component to prepare lipid-based nanoparticles, as this is expected to increase the skin deposition and enhance the localized photo-cytotoxicity of Cur allowing skin cancer targeting.

Solid lipid nanoparticles of various lipid components have been studied as nanocarriers for Cur in previous literatures^14,15^. Another recent study has performed surface modification to improve Cur bioavailability^16^.

However, the novel point in this study is focused on the nanoencapsulation of Cur using a PEGylated lipid-based nanosystem for skin targeting; different surfactants were investigated. The designed Cur-loaded PEGylated lipid nanocarrier (PLN) was evaluated and examined for dark- and photo-cytotoxicity against human epidermoid squamous cell carcinoma cell line (A431). In-vivo skin deposition and histopathological studies were also included in the study.

## Methodology

### Materials

Tefose 1500 (T1500) was obtained from Gattefosse (St Priest, France). Span 85 (S85), Span 20 (S20), Tween 80 (T80), and Tween 20 (T20) were purchased from Sigma Chemical Company (St. Louis, Missouri, USA). Curcumin (Cur) purity 95% was purchased from Sigma Aldrich, Germany. Visking Dialysis tubing, diameter 21 mm was purchased from Serva electrophoresis, Germany. All other chemicals used were of analytical grade.

For cell culture, human epidermoid squamous cell carcinoma cell line (A431) was obtained from American tissue culture collection (ATCC) through the bioassay-cell culture laboratory, National Research Centre, Egypt. Cells were suspended in DMEM-F12 medium (purchased from Sigma) with 10% foetal bovine serum (FBS), 1% mixture of 10,000 U/ml potassium penicillin, 10,000 µg/ml streptomycin sulfate, and 25 µg/ml amphotericin B (all were purchased from Lonza, Belgium) and incubated at 37 °C under 5% CO_2_ using a water jacketed CO_2_ incubator (Sheldon, TC2323, Cornelius, OR, USA) and regularly checked for absence of any contamination. For MTT assay, 3-(4,5-dimethylthiazol-2-yl)-2,5-diphenyl tetrazolium bromide (MTT) was purchased from BIO BASIC CANADA (Ontario, Canada) and Sodium dodecyl sulfate (SDS) was purchased from ADWIC, Egypt.

### Methods

#### Preparation of the Cur-loaded PEGylated lipid nanocarriers (PLN)

A previously described method was followed to prepare the Cur-loaded PEGylated lipid nanocarriers (PLN), where a coarse emulsion was produced followed by probe sonication using Sonifier Model 250 (Branson Ultrasonics, USA)^10,11,17^. Briefly, the drug (10 mg/g) together with the lipophilic component (Tefose 1500) were mixed to form the lipid phase which was melted at 50 °C. The aqueous phase was heated to the same temperature and then, it was added to the oily phase to form a coarse emulsion which was subjected to probe sonication at 20 W for 90 s. As listed in Table 1, different surfactants were used namely: Span 85 (S85), Span 20 (S20), Tween 80 (T80) and Tween 20 (T20). The final preparation was kept in amber glass vial and kept at room temperature till use.

**Table 1:** Composition, HLB, particle size analysis (PS, PDI &Z) and in-vitro release of the Cur-loaded PEGylated lipid nanocarriers (PLN).

Formula	Surfactant	HLB value	E.E	PS (nm)	PDI	Z	Q after 24 h	Q after 48 h
PLN1	S85	1.8	35.51 ± 2.05	520.00 ± 25.23	1 ± 0.09	− 35.90	15.01 ± 3.01	20.45 ± 4.21
PLN2	S20	8.6	38.00 ± 3.04	260.01 ± 24.63	0.513 ± 0.03	− 31.71	5.52 ± 1.51	7.52 ± 2.65
PLN3	T80	15	40.01 ± 2.10	167.60 ± 15.12	0.323 ± 0.02	− 26.91	75.02 ± 4.61	98.25 ± 6.89
PLN4	T20	16.7	40.02 ± 2.11	182.00 ± 15.56	0.325 ± 0.03	− 17.92	40.10 ± 4.00	50.20 ± 4.62

All values present the mean ± SD (n = 3).

Hydrophilic lipophilic balance (HLB)/average particle size (PS)/polydispersity index (PDI)/zeta potential (Z)/encapsulation efficiency (EE)/cumulative amount of drug release % (Q) after 24 h or 48 h.

Span 85 (S85)/Span 20 (S20)/Tween 80 (T80)/Tween 20 (T20).

#### Characterization and evaluation of the Cur-loaded PEGylated lipid nanocarriers (PLN)

##### Encapsulation efficiency

The un-entrapped drug was separated from the entrapped by centrifugation for 30 min, at 10,000 rpm at 8 °C (Centrikon T-42K, Kontron, Instruments, UK). The precipitated loaded nanoparticles were then dissolved in ethanol and Cur concentration was measured spectrophotometrically at 420 nm by UV–VIS double beam spectrophotometer (Rayleigh UV-2601)^18^. The encapsulation efficiency (EE) was calculated as a ratio of the initially added drug amount.

##### Particle size analysis and zeta potential

Mean particle size, size distribution and zeta potential measurements were performed using the Malvern Zetasizer Nano ZS (Malvern Instruments Ltd., Malvern, UK) by photon correlation spectroscopy (PCS). Before measurement, samples were diluted with distilled water appropriately.

##### In-vitro drug release study

Samples (100 mg) from each formula were accurately weighed and placed in a dialysis membrane (molecular weight cut off 12,000–14,000). To allow sink conditions, the dialysis membranes was immersed in 50 ml PBS buffer (pH 7.4) containing 10% ethanol as a receptor medium, and kept under stirring (100 rpm) at 37 °C. Aliqouts of 1 ml were withdrawn at different time intervals up to 48 h and replaced by fresh medium. The concentration of Cur in the withdrawn samples were measured spectrophotometrically at 420 nm.

#### Characterization of the selected Cur-loaded PEGylated lipid nanocarriers (PLN3)

##### Differential scanning colorimetry

Differential scanning calorimetry (DSC) analysis was carried out using DSC 60 (Shimadzu, Japan). The free drug, the drug-loaded nanoparticles and individual components of the nanoparticles were placed in aluminium pans. The temperature was increased from 25 to 200 °C at a rate of 10 °C/min under nitrogen atmosphere.

##### Transmission electron microscopy (TEM)

A diluted colloidal suspension of the sample was spread on a carbon-coated copper grid without staining prior to examination by transmission electron microscope (JEM 100S, Jeol, Ltd., Tokyo, Japan).

#### In-vitro cytotoxicity

Cells suspended in fresh medium were seeded at a concentration of 1 × 10^4^ cells/well in 96-well microtiter plastic plates and incubated for 24 h till complete attachment. Afterwards, the cells were incubated in dark for 24 h either in the fresh media alone (negative control) or with different concentrations (20, 10, 5, 2.5 and 1 μg/ml) of the tested samples: free Cur suspension (Cur) and the selected Cur-loaded PEGylated lipid nanocarriers (PLN3). To investigate the PDT effect on the cytotoxicity, some of the cells, after incubation with tested samples, were irradiated at a fluence of 300 mW/cm^2^ for 4 min by blue light delivered from a non-coherent light 200 W halogen lamp (Photon scientific, Cairo, Egypt) mounted in a housing cooled by fans. The light emitted passed through glass filter containing circulating water for omission of ultraviolet and infrared radiation then through a band pass filter (Rosco Laboratory, Ltd., Stamford) with transmission spectral range of 320–540 nm and maximum transmission at 410 nm^18^.

Finally, the dark toxicity (after incubation with the tested samples) and the phototoxicity (24 h post irradiation) was assessed by MTT assay. Briefly, the medium was washed out, 40 μl MTT solution (2.5 μg/ml) were added to each well and incubated for further 4 h at 37 °C under 5% CO_2_. To dissolve the formed crystals, 200 μl of 10% Sodium dodecyl sulfate (SDS) in deionized water was added to each well and incubated overnight at 37 °C. The absorbance was then measured using a microplate multi-well reader (Bio-Rad Laboratories Inc., model 3350, Hercules, California, USA) at 595 nm and a reference wavelength of 620 nm.

#### In vivo studies

##### Animals

Male *Mus musculus* Albino mice (23 ± 2 g, 7 weeks old) were supplied from the animal house of National Research Centre, Egypt and they were kept there for 24 h in clean plastic well ventillated cages under standard laboratory conditions with free access to food and water. The study protocol was approved by Cairo University Institutional Animal Care and Use Committee (CUIACUC), approval number (CU-I-F-31–19). All experiment protocol was performed in accordance with relevant guidelines and regulations set by the institutional committee.

##### Experiment

The hair on the dorsal skin was removed by razor. Afterwards, the samples (100 mg of PLN3 or Cur suspension containing an equivalent amount of the drug) were topically applied on 1 cm^2^ area of the shaved dorsal skin. The animals were randomly subdivided into different groups so that each group contained six animals, as following:

- Control negative: animals did not receive any treatment and served as control.
- Control-light: animals were irradiated but didn't receive any treatment.
- Group A: animals were topically treated by Cur aqueous suspension.
- Group B: animals were topically treated by Cur suspension, followed by irradiation after 1 h.
- Group C: animals were topically treated by the selected Cur-loaded PEGylated lipid nanocarrier (PLN3).
- Group D: animals were topically treated by PLN3, followed by irradiation after 1 h.

The irradiated groups were exposed to blue light delivered by light emitting diode LED (420 nm) for 10 min at a fluence of 90 mW/cm^2^ (Photon scientific, Cairo, Egypt). The fluence was adjusted using a powermeter (Gentec-solo PE, Canada). During irradiation, the animal behavior was monitored, and animals showed any signs of pain or distress were subjected to very low doses of isoflurane inhalation.

Animals were kept separately in clean plastic cages (one animal in each cage) for 24 h. Afterwards; execution of animals was done by cervical dislocation under anesthesia and the treated skin areas were separated by scissors.

##### Skin deposition

The separated dorsal skin were accurately weighed, cut into small pieces and homogenized in ethanol in order to extract the Cur from the skin. The extract was centrifuged at 5,000 rpm for 10 min, and the content of Cur in the supernatant was measured spectrophotometrically at 420 nm as mentioned above.

##### Histopathology

The separated dorsal skin from all groups was fixed in 10% formalin for 24 h. Then, they were washed and dehydrated by serial dilutions of alcohol. Paraffin bees wax tissue blocks (4 µm thickness) were prepared by slide microtome. The obtained tissue sections were collected on glass slides, deparaffinized, stained by hematoxylin and eosin and examined under light microscope.

##### Confocal laser scanning microscopy (CLSM)

The separated skin sections from negative control group, group A and group C were treated as above without staining the glass slides. The unstained slides were examined by confocal laser scanning microscope (LSM 710, Carl Zeiss, ZEISS), at excitation wavelength of 405 nm and the images were analyzed for fluorescence intensity using Zen 2009 software version.

### Statistical analysis

Data are expressed as the mean of three experiments ± the standard deviation (SD) and were analyzed using one-way analysis of variance, followed by the least significant difference procedure using SPSS software (SPSS, Inc., Chicago, Illinois, USA). Statistical differences yielding p < 0.05 were considered significant.

## Results and discussion

### Characterization and evaluation of the prepared formulae

#### Drug encapsulation efficiency (EE)

Table 1 is showing the drug encapsulation efficiency results, the surfactant used in the preparation didn't have an effect on this parameter, therefore all the PLN have an EE value ranging from 35.51 to 40.02% and there was no significant difference between them (p  > 0.05).

#### Particle size analysis

As listed in Table 1, it is clear that the PLN prepared using the Tweens (PLN3 and PLN4) had a smaller particle size than those prepared using the Spans (PLN1 and PLN2) (p < 0.05). This may be due to the higher HLB in case of the former as it was previously reported that the HLB value influence the formation and properties of lipid-based nanoparticles^19,20^. A previous study has reported that surfactants of lower lipophilicity formed nanovesicles with smaller sizes^20^.

Table 1 is showing the PDI values, Spans-containing PLN had higher PDI values than Tweens-containing PLN. Low PDI values are recommended (< 0.5) as an indication of a uniform distribution and low aggregation possibility.

The Z potential values are also listed in Table 1; the values are − 35.90, − 31.71, − 26.91 and − 17.92 for PLN1, PLN2, PLN3 and PLN4, respectively. The range of reported values is considered to be high enough to ensure satisfactory physical stability with a low aggregation tendency due to electrostatic repulsion between the particles^21,22^. The use of PEGylated lipids can help to enhance the stability and prevent the agglomeration of the prepared nanoparticles due to the formation of the polymeric-coated nanoparticles advantageous for drug delivery^13,23^.

#### In-vitro drug release study

**Figure 1:**
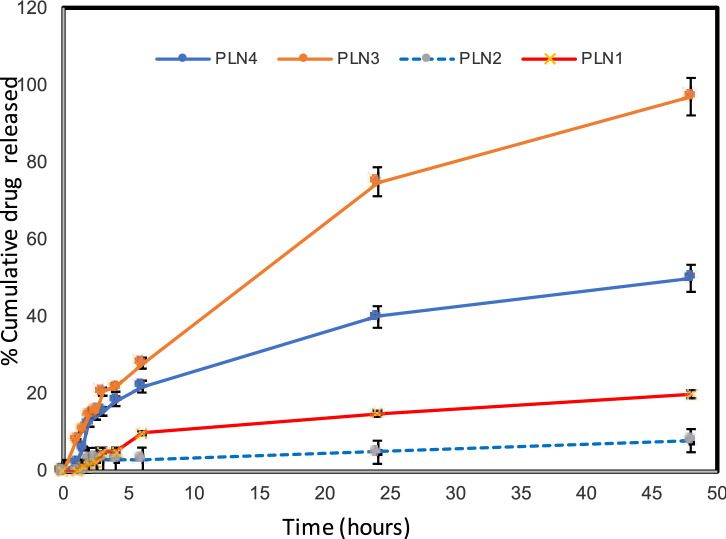
In-vitro drug release profiles of curcumin from the prepared PLN using dialysis membrane.

Curcumin release pattern from the prepared PLN is shown in Fig. 1 and release data are listed in Table 1. It is clear that Tweens-containing PLN attained faster release than Spans-containing PLN (p < 0.05); this runs in agreement with the results discussed above regarding the smaller particle sizes of the former; besides their higher hydrophilicity which allows for a more rapid release. PLN3 had the fastest drug release profile (p < 0.05) and showed a complete drug release at 48 h. The cumulative amount of drug release (%) was 75.02 ± 4.61 and 98.25 ± 6.89 at 24 and 48 h, respectively.

Based on the above studies, PLN3 was selected for further investigations. These nanoparticles had the smallest particle size with uniform distribution and attained the fastest drug release pattern.

#### Transmission electron microscopy

**Figure 2:**
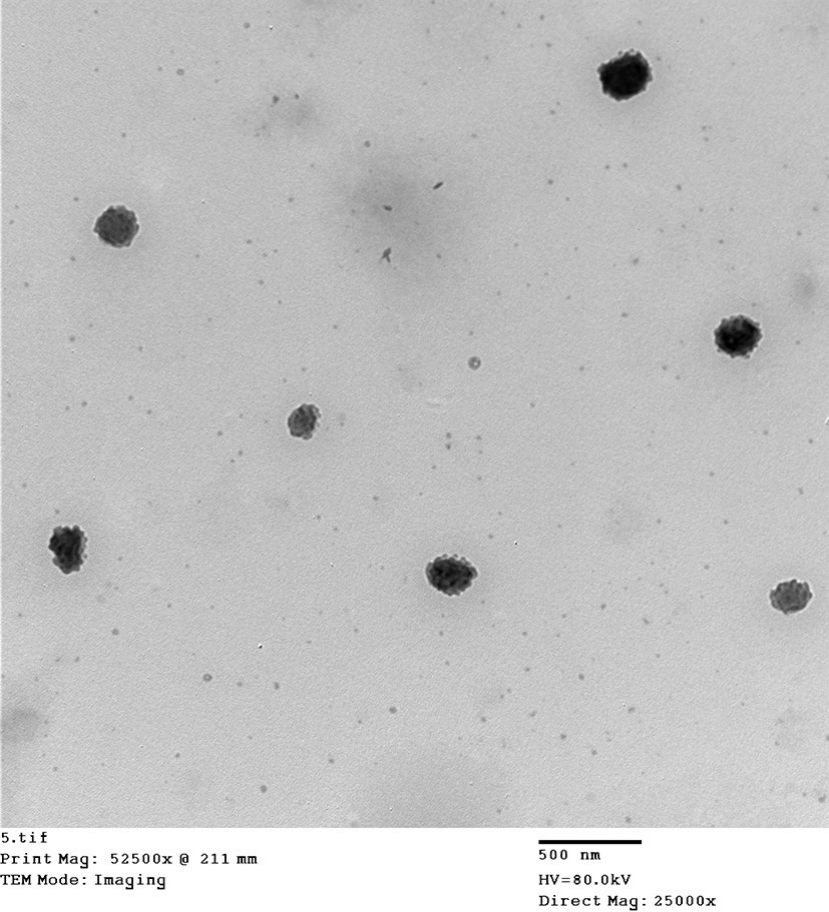
TEM photograph of the selected Cur-loaded PEGylated lipidic nanoparticles (PLN3).

The photograph of the selected PLN (PLN3) is shown in Fig. 2. The nanoparticles are spherical and well-separated with a particle size range correlating with that recorded in the particle size analysis. The core–shell nanostructural architecture can be observed in the photo, this runs in agree with a previous study pointing out to the presence of a solid core surrounded by a PEG coating in such a type of nanocarriers^13^.

#### Differential scanning calorimetry

**Figure 3:**
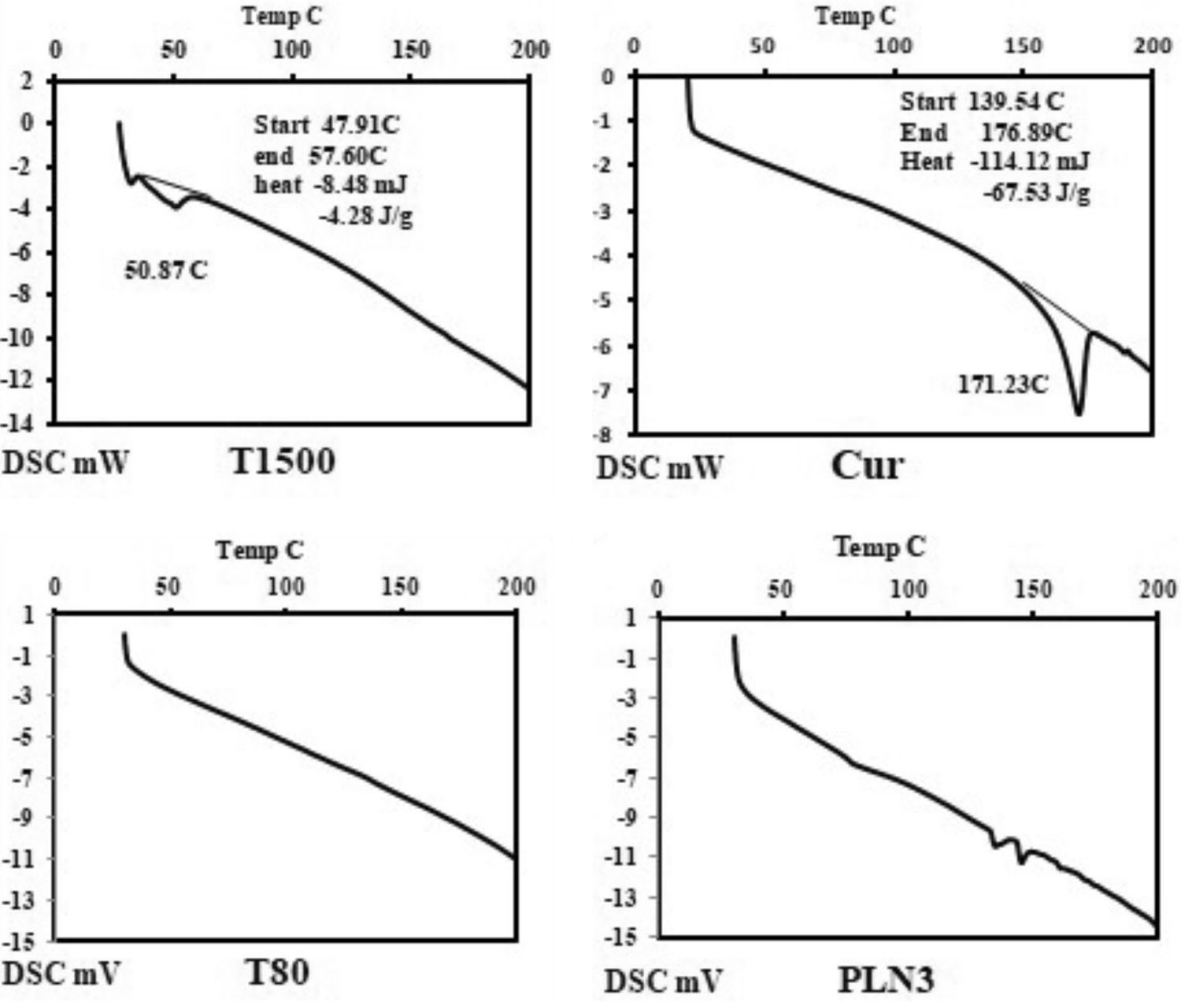
DSC thermograms of the selected Cur-loaded PEGylated lipidic nanoparticles (PLN3) and its individual components: Tefose 1500 (T1500), Tween 80 (T80), and the drug (Cur).

The thermograms of the selected preparation (PLN3) and its individual components are illustrated in Fig. 3. The used surfactant (T80) did not show transition endothermic peaks within the tested temperature range (from 25 to 200 °C) while the drug (Cur) and the lipid (T1500) showed endothermic peaks corresponding to their melting points at 171.23 °C and 50.86 °C, respectively. The mentioned characteristic peaks were not observed in the lipid nanoparticles thermogram, which can indicate the absence of the crystalline state of the drug and its entrapment within the lipid core of the formulated lipid-based nanoparticles.

### In vitro cytotoxicity

**Figure 4:**
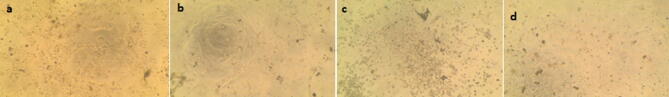
Photographs of cytotoxicity on human epidermoid squamous cell carcinoma cell line (A431) at Cur concentration equivalent to 20 µg/ml: (**a**) PLN3 in dark, (**b**) Cur in dark, (**c**) PLN3 after irradiation and (**d**) Cur after irradiation. *Cur* curcumin suspension, *PLN3* Cur-loaded PEGylated lipidic nanoparticles.

The dark- and photo-cytotoxicity of free Cur suspension and PLN3 were assessed on a human epidermoid squamous cell carcinoma cell line (A431). Visual inspection of the cells under inverted microscope proved the efficacy of the tested samples, cells of all groups treated with the highest drug dose (equivalent to 20 μg/ml) are detached and dead (Fig. 4).

**Figure 5:**
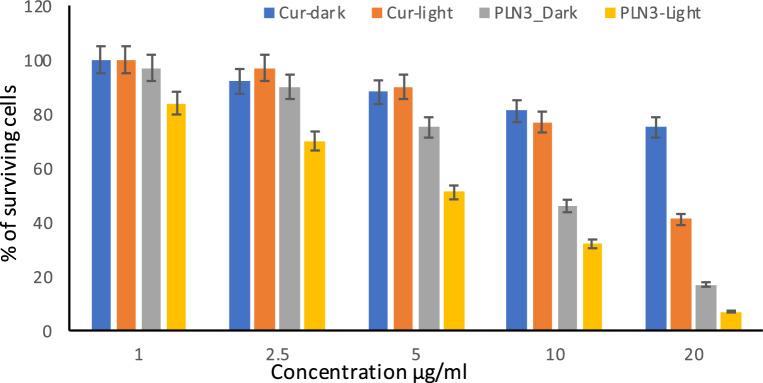
Cytotoxicity of different concentrations of the Cur suspension (Cur) and of the selected Cur-loaded PEGylated lipidic nanoparticles (PLN3) on human epidermoid squamous cell carcinoma cell line (A431) in dark and after irradiation.

MTT assay results (Fig. 5) revealed that the cytotoxicity of Cur and PLN3 were concentration dependent. The higher efficacy of the designed Cur-loaded nanoparticles (PLN3) compared to Cur suspension is obvious at all studied concentrations in dark as well as light conditions. The viability of the cells treated with PLN3 was significantly lower (p < 0.05) than those treated by free Cur suspension. Moreover, the cell viability of cells treated by PLN3 at all concentrations was significantly decreased upon light radiation (p < 0.05).

At concentration of 20, 10, 5, 2.5, and 1 μg/ml, the % of surviving cells was found to be 17, 46, 75, 90, and 97%, respectively for PLN3 compared to 75, 81, 88, 92, and 100%, respectively for Cur suspension in dark conditions. While upon irradiation, % of surviving cells was found to be 7, 32, 51, 70, and 84%, respectively for PLN3 compared to 41, 77, 90, 97 and, 100%, respectively for Cur suspension.

These results might prove that Cur can induce cytotoxicity which can be enhanced upon loading it in a suitable nanocarrier, and can be further increased upon exposure to irradiation. Nanocarriers can enhance the Cur cytotoxicity due to the drug solubilization, increase of surface area, enhancement of permeability and cellular uptake. Upon exposure to the blue light (430 nm), the cytotoxicity was greatly enhanced due to the generation of reactive oxygen species (ROS) that damage the cellular organelles and disrupt the mitochondrial membrane integrity leading to apoptosis^14,18,24^.

After irradiation, curcumin and demethoxycurcumin were reported to induce apoptosis through mitochondrial pathways, this effect was studied in details on keratinocyte cell line (HaCat) and squamous cell carcinoma (A431)^4,25^.

In this study, we designed PEGylated lipid nanoparticles to be explored as Cur carrier for improvement of skin targeting; the designed nanosystems were found to be superior to Cur suspension in inducing cytotoxicity. Moreover, the above-mentioned studies used UVB as a light source to excite Cur. Instead, in this study we used a cheaper, safer and readily manufactured visible light source emitting blue light; the obtained results proved that it induced photodynamic effect efficiently. This light source was used previously to induce photo-toxicity of Cur on HePg2 cells^18^.

### In Vivo studies

#### Skin deposition

The amount of Cur extracted from the skin of groups treated by PLN3 was approximately twice that of groups treated by aqueous Cur suspension (Table 2). This significant difference (p < 0.05) could be attributed to the unique features of the prepared PEGylated lipidic nanoparticles which promote penetration and accumulation of the drug in the skin layers. These results confirm the expectation which pushed us to conduct this study, as Yuan et al. reported that PEGylated solid lipid nanoparticles enhanced the oral bioavailability^26^. Furthermore, it was proved that association of PEG with the lipid nanoparticles prevented their aggregation and decreased enzymatic degradation in gastrointestinal fluids, this hypothesized that PEG-stearate formed a stabilizing/enzyme repellent coating on the lipid nanoparticles^27^. Another study proved that polymeric-coated nanoparticles showed a higher drug retention and lower clearance at the site of administration compared to the uncoated nanoparticles^23^. Our results provide additional beneficial effect of PEGylated lipid nanocarriers in dermal delivery as they were able to improve the skin penetration and deposition of Cur. However, the light didn’t affect the deposition of Cur in skin layers.

**Table 2:** Amount of Cur deposited in skin in different animal groups.

Animal group	Amount of Cur (µg/g skin)
Group A	2.7 ± 0.33
Group B	2.8 ± 0.40
Group C	5.7 ± 0.35
Group D	5.3 ± 0.20

All values present the mean ± SD (n = 3).

Group A: animals were topically treated by Cur aqueous suspension.

Group B: animals were topically treated by Cur suspension, followed by irradiation after 1 h.

Group C: animals were topically treated by 100 mg of the selected Cur-loaded PEGylated lipid nanocarrier (PLN3).

Group D: animals were topically treated by 100 mg of the selected Cur-loaded PEGylated lipid nanocarriers (PLN3), followed by irradiation after 1 h.

#### Confocal laser scanning microscopy

**Figure 6:**
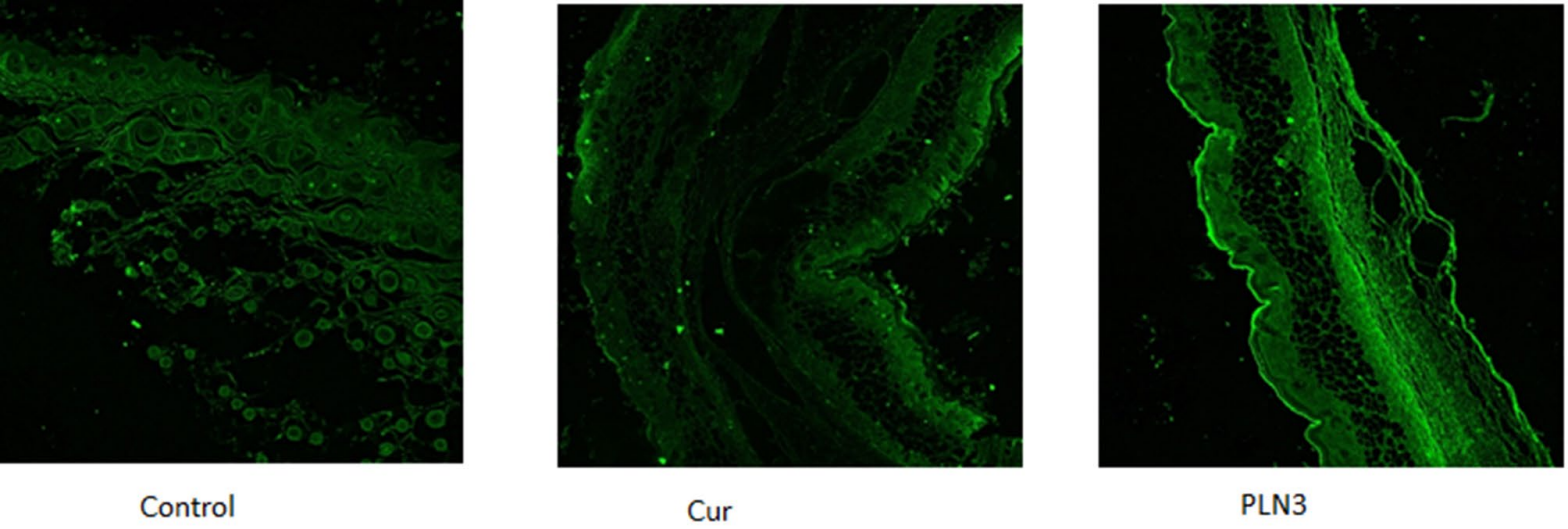
Confocal laser scanning microscopy photographs of dorsal mice skin of control, group treated with drug suspension (Cur) and group treated with the selected Cur-loaded PEGylated lipidic nanoparticles (PLN3).

Confocal microscopy was used to visualize the fluorescence of delivered Cur across the skin. Cur is a fluorescent molecule that emits fluorescence upon excitation by blue light, however, at this excitation wavelength the untreated mice skin was found to produce auto-fluorescence due to the presence of endogenous fluorophores such as elastin and collagen^28^. Therefore, the confocal images (Fig. 6) obtained for skin sections treated by Cur suspension and PLN3 were compared to those of untreated skin of the control group in terms of fluorescence intensity. The fluorescence intensity was increased by about 260% and 480% in case of Cur suspension and PLN3, respectively. The enhancement of fluorescence intensity indicates that encapsulating Cur in the suggested PEGylated lipidic nanocarrier has improved its penetration and accumulation into the skin. These results run in accordance with all the above-mentioned results regarding the better tissue penetration and deposition of the designed carrier.

#### Histopathological findings

**Figure 7:**
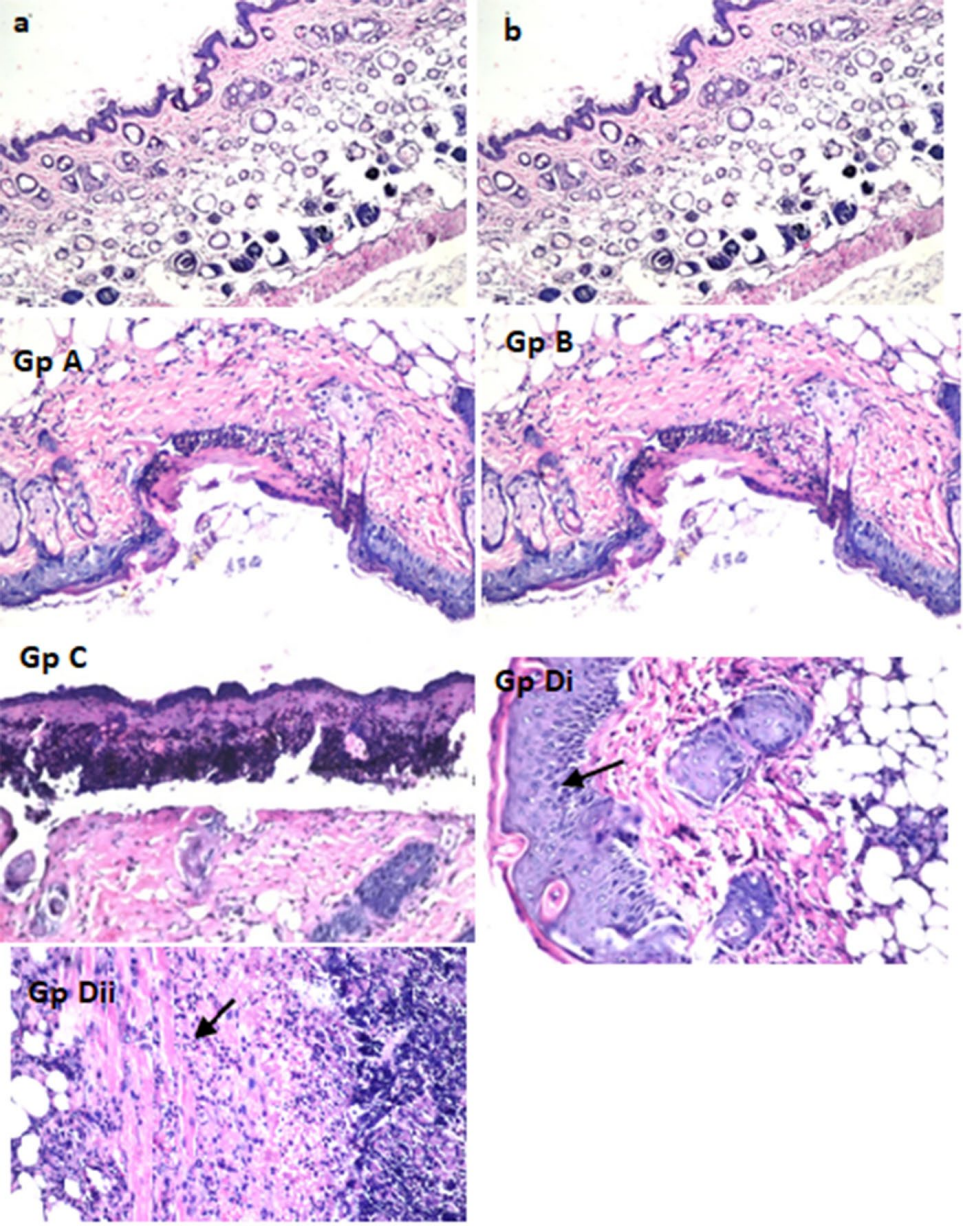
Transverse stained (H&E) sections of the dorsal mice skin taken from different groups: (**a**) negative control, (**b**) irradiated control, GpA: animals were topically treated by Cur aqueous suspension, Gp B: animals were topically treated by Cur suspension, followed by irradiation after 1 h, Gp C: animals were topically treated by 100 mg of the selected Cur-loaded PEGylated lipid nanocarrier (PLN3), GpDi animals were topically treated by 100 mg of the selected Cur-loaded PEGylated lipid nanocarriers (PLN3), followed by irradiation after 1 h showing the acanthosis (increasing thickness of the epidermal layer, indicated by the arrow) and GpDii the subcutaneous tissues of the same group with focal inflammatory cells infiltration (indicated by the arrow).

Histopathological examination recorded the changes in the structure of different skin layers in different groups as illustrated in Fig. 7.

No histological changes were observed in all skin layers of the negative control and the irradiated control groups (Fig. 7a,b respectively).

In group A, treated by aqueous Cur suspension, the epidermis and the underlying superficial layers of dermis exhibited areas of focal ulceration and necrosis, due to the interaction between Cur and the skin surface. The areas of epidermal ulceration and necrosis were wider and more obvious in group C treated by PLN3, because the PEGylated lipidic nanoparticles delivered and deposited a higher amount of the drug into the skin as revealed from the above-mentioned results concerning cytotoxicity and skin deposition. Moreover, the interaction between the lipids of the formula and the skin lipids may lead to disruption of the lamellar arrangement of the epidermis^29^. The effect on the epidermal layer was exaggerated after radiation with blue light as indicated by acanthosis (increasing skin thickness) of the epidermal layer noticed in the irradiated groups (group B and D) as shown in Fig. 7.

The subcutaneous tissues and musculature showed focal inflammatory cells infiltration in all groups. However, in the groups treated by PLN3 in presence or absence of light (groups C and D), the inflammatory cells infiltration was massive and associated with aggregation (Fig. 7, Group Dii).

From these finding we can conclude that the prepared PLN3 could increase the penetration and deposition of Cur into skin layers, intensifying and extending its effect to reach deeper skin layers, in addition to its stabilizing effect. Consequently, it potentiates the efficacy of Cur as a photosensitizer in photodynamic therapy of skin cancer.

## Conclusion

This study proves the positive impact of the use of PEG-grafted pharmaceutical ingredients. The displayed results show the feasibility of a targeted and enhanced photodynamic therapy of skin carcinoma using Cur-loaded PEGylated lipidic nanoparticles. This can shed the light on a promising, safe, economic and effective method to fight cancer.
